# Downgraded Lymphoma: B-Chronic Lymphocytic Leukemia in a Known Case of Diffuse Large B-Cell Lymphoma - De Novo Occurrence or Transformation

**DOI:** 10.4274/tjh.2015.0164

**Published:** 2015-12-03

**Authors:** Smeeta Gajendra, Bhawna Jha, Shalini Goel, Tushar Sahni, Pranav Dorwal, Ritesh Sachdev

**Affiliations:** 1 Medanta-The Medicity, Department of Pathology and Laboratory Medicine, Gurgaon, India

**Keywords:** Diffuse large B-cell lymphoma, Chronic lymphocytic leukemia, Downgraded lymphoma

## TO THE EDITOR

Low-grade indolent lymphomas can be transformed into high-grade aggressive lymphomas [1,2,3,4]. Very few cases of transformation of high/intermediate-grade lymphoma to low-grade lymphoma have been reported in the literature [5,6]. This may arise through transformation of the original clone or may represent a new neoplasm resulting from additional genetic mutations that alter the growth rate, growth pattern, and sensitivity to treatment.

**Figure 1:**
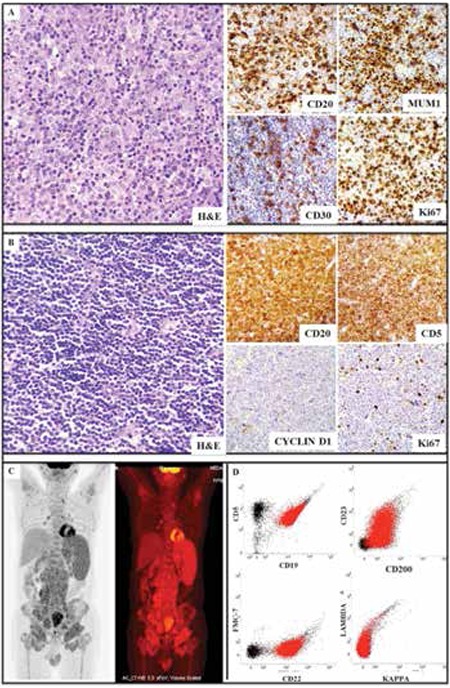
A) Lymph node biopsy showing diffuse infiltration of large atypical cells with prominent nucleoli and vesicular chromatin, which were positive for CD20, CD30, and MUM1 with a Ki67 index of 80%. B) Biopsy from paraaortic mass showing small-sized neoplastic cells with scant cytoplasm, hyperchromatic nuclei, and clumped chromatin arranged in sheets, which were positive for CD20 and CD5 and negative for cyclin D1 with a low Ki67 index. C) 18F-FDG PET-CT showing multiple cervical, supraclavicular, mediastinal, axillary, abdominal, and pelvic lymphadenopathies with gross splenomegaly. D) Immunophenotyping of peripheral blood smear showing 84% abnormal lymphoid cells, which were positive for CD19, CD5, CD23, CD22 (dim), and CD200 with lambda light chain restriction and negative for FMC7.

 A 57-year-old male diagnosed with diffuse large B-cell lymphoma (DLBCL) (non-germinal center B-cell type) in 2002 completed 6 cycles of CHOP followed by radiotherapy. In 2006, 18F- fluorodeoxyglucose (FDG) positron emission tomography/computed tomography (PET/CT) showed no active disease. In 2007 there was recurrence in the left obturator and external iliac nodes. Lymph node biopsy done outside our facility showed CD20+ B-cell lymphoma. The patient was advised to undergo intensive chemotherapy, but was lost to follow-up. In 2010, the patient came to our hospital with bilateral firm non-tender inguinal and right axillary lymphadenopathy without any organomegaly. 18F PET/CT revealed heterogeneous uptake in the left paraaortic, retrocaval, precaval, and bilateral internal iliac nodes. A previous diagnostic lymph node biopsy was reviewed, showing diffuse infiltration of large atypical cells, positive for CD20, CD30, MUM1, and Bcl2 with a Ki67 index of 80% and negative for CD3, CD5, and CD10, which was consistent with DLBCL (Figure 1A). Biopsy of the paraaortic mass revealed sheets of small lymphoid cells, which were positive for CD20, CD5, and CD23 and negative for CD3 and cyclin D1 with a low Ki67 index, suggestive of small-cell lymphoma (Figure 1B). 18F PET-CT was repeated after 1 year, showing multiple FDG-avid cervical, supraclavicular, mediastinal, axillary, abdominal, and pelvic lymphadenopathies (Figure 1C). After 10 months, hemoglobin was 90 g/L, total leukocyte count was 21.1x109/L, and platelet count was 40x109/L. Peripheral blood smear showed 84% abnormal lymphoid cells, which were immunopositive for CD19, CD5, CD23, CD22 (dim), CD200, and CD20 with lambda light chain restriction and negative for CD10, FMC7, CD38, IgM, and CD103, confirming the diagnosis of chronic lymphocytic leukemia (CLL) (Figure 1D). The patient was started on a fludarabine, cyclophosphamide, and rituximab (FCR) regimen. After 6 cycles of FCR, he was in complete remission and was started on rituximab maintenance therapy.

The phenomenon of high- or intermediate-grade non-Hodgkin lymphoma recurring as a low-grade lymphoma is an uncommon form of transformation known as “downgraded” lymphoma. This downgrading may be due to: 1) recurrence of a low-grade lymphoma that was present as a minor component of the initial lymphoma or in a site not biopsied, or 2) development of a second lymphoma resulting from chemotherapy and/or an intrinsic propensity for lymphoma development in the patient [5,6]. Relapse in DLBCL mainly occurs in the first 2 to 3 years, while late relapses after 5 years are rare, occurring in 3.6% of cases. Patients with DLBCL relapse usually have the same histology. However, relapse as indolent lymphoma following initial DLBCL may occur in about 17% of cases, predominantly as follicular lymphoma or rarely as nodal marginal zone lymphoma or as extranodal mucosa-associated lymphoid tissue lymphoma [7]. Histopathological examination including extensive immunohistochemistry should be done, not only when transformation is clinically suspected but also at each recurrence because the disease can recur as indolent lymphoma and an accurate histologic diagnosis will contribute to a better understanding of the pathogenesis of transformation and the start of prompt therapy to improve the survival of the patients.
